# Supplementary material for: Transcriptome Analysis of Human Endogenous Retroviruses at Locus-Specific Resolution in Non-Small Cell Lung Cancer
Source: Cancers (Basel). 2022 Sep 13;14(18):4433. doi: 10.3390/cancers14184433 (PMC9497127; doi:10.3390/cancers14184433)
Supplement: Supplementary file 1 [file cancers-14-04433-s001.zip › figure_S1.pdf]

Fig. S1

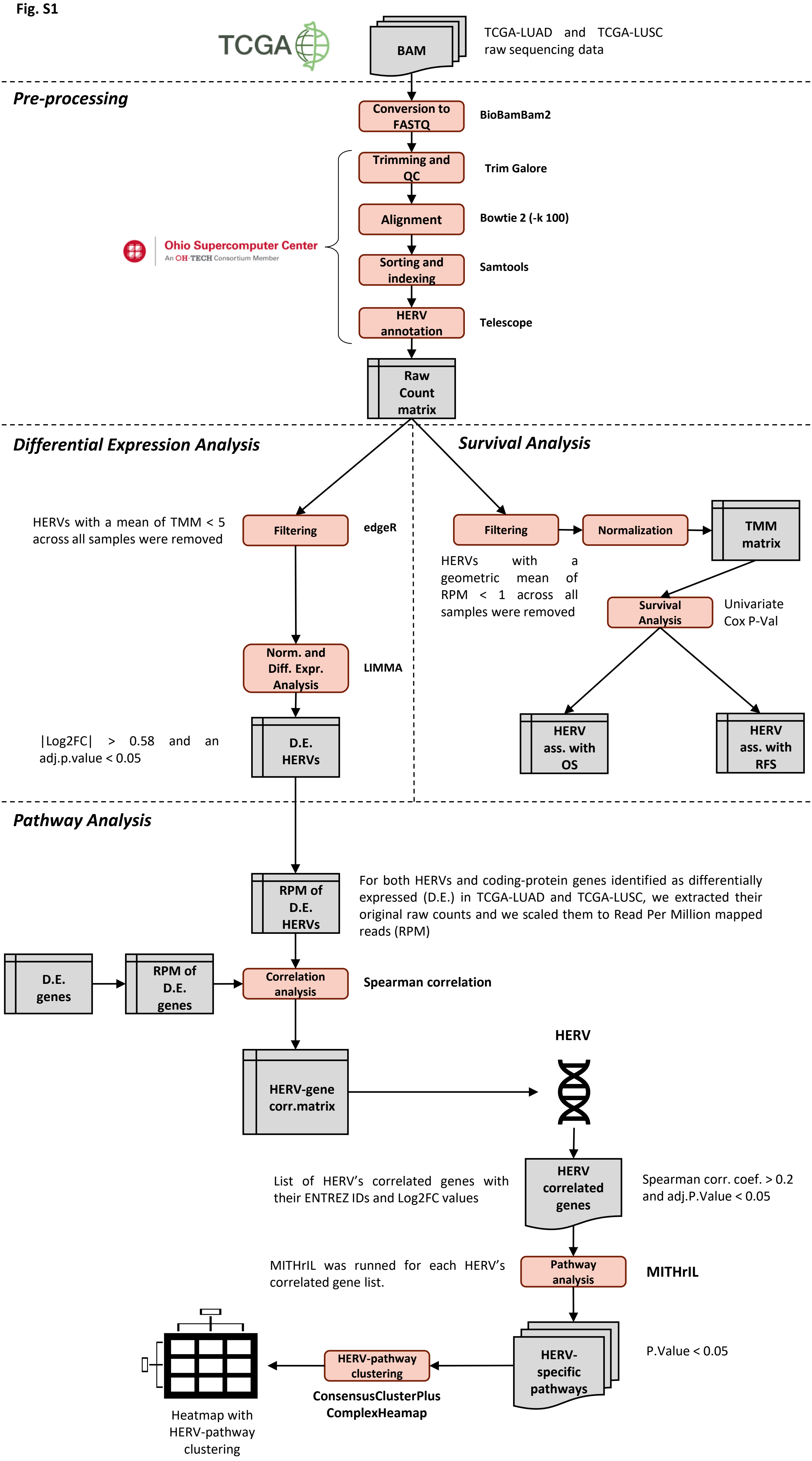
TCGA
BAM
TCGA-LUAD and TCGA-LUSC raw sequencing data
Pre-processing
Conversion to FASTQ
BioBamBam2
Trimming and QC
Trim Galore
Alignment
Bowtie 2 (-k 100)
Ohio Supercomputer Center
An OH·TECH Consortium Member
Sorting and indexing
Samtools
HERV annotation
Telescope
Raw Count matrix
Differential Expression Analysis
Survival Analysis
HERVs with a mean of TMM < 5 across all samples were removed
Filtering
edgeR
Filtering
Normalization
TMM matrix
HERVs with a geometric mean of RPM < 1 across all samples were removed
Survival Analysis
Univariate Cox P-Val
Norm. and Diff. Expr. Analysis
LIMMA
|Log2FC| > 0.58 and an adj.p.value < 0.05
D.E. HERVs
HERV ass. with OS
HERV ass. with RFS
Pathway Analysis
RPM of D.E. HERVs
For both HERVs and coding-protein genes identified as differentially expressed (D.E.) in TCGA-LUAD and TCGA-LUSC, we extracted their original raw counts and we scaled them to Read Per Million mapped reads (RPM)
D.E. genes
RPM of D.E. genes
Correlation analysis
Spearman correlation
HERV
HERV-gene corr.matrix
List of HERV's correlated genes with their ENTREZ IDs and Log2FC values
HERV correlated genes
Spearman corr. coef. > 0.2 and adj.P.Value < 0.05
MITHrIL was runned for each HERV's correlated gene list.
Pathway analysis
MITHrIL
HERV-specific pathways
P.Value < 0.05
HERV-pathway clustering
ConsensusClusterPlus
ComplexHeamap
Heatmap with HERV-pathway clustering
