# Supplementary material for: Transcriptome Analysis of Human Endogenous Retroviruses at Locus-Specific Resolution in Non-Small Cell Lung Cancer
Source: Cancers (Basel). 2022 Sep 13;14(18):4433. doi: 10.3390/cancers14184433 (PMC9497127; doi:10.3390/cancers14184433)
Supplement: Supplementary file 1 [file cancers-14-04433-s001.zip › Table_S1.pdf]

LUAD				LUSC			
HERV SUB-FAMILY	N. Expr HERVs	N. HERV family	Perc. Expr HERV family	HERV SUB-FAMILY	N. Expr HERVs	N. HERV family	Perc. Expr HERV family
**HERVH**	575	1206	47,7	**HERVH**	651	1206	54
**ERVL-E**	438	2240	19,5	**ERVL-E**	449	2240	20
**HERVK**	366	841	43,5	**ERV3-16A3_I**	369	1983	18,6
**ERV3-16A3_I**	350	1983	17,6	**HERVK**	363	841	43,1
**HERVL**	245	1082	22,6	**HERVL**	259	1082	24
**ERVL-B4**	236	1206	19,5	**ERVL-B4**	245	1206	20,3
**MER4**	218	609	35,8	**MER4**	235	609	38,6
**MER41**	149	442	33,7	**MER41**	152	442	34,4
**MER61**	112	355	31,5	**MER61**	115	355	32,4
**HERV17**	100	227	44,1	**HERV17**	101	227	44,5
