# Supplementary material for: Transcriptome Analysis of Human Endogenous Retroviruses at Locus-Specific Resolution in Non-Small Cell Lung Cancer
Source: Cancers (Basel). 2022 Sep 13;14(18):4433. doi: 10.3390/cancers14184433 (PMC9497127; doi:10.3390/cancers14184433)
Supplement: Supplementary file 1 [file cancers-14-04433-s001.zip › Table_S2.pdf]

LUAD				LUSC			
HERV SUB-FAMILY	N. DE HERVs	N. HERV family	Perc. DE HERV family	HERV SUB-FAMILY	N. DE HERVs	N. HERV family	Perc. DE HERV family
**HERVH**	236	1206	19,5	**HERVH**	378	1206	31,3
**ERVL-E**	148	2240	6,6	**ERV3-16A3_I**	228	1983	11,5
**HERVK**	136	841	16,2	**ERVL-E**	228	2240	10,2
**ERV3-16A3_I**	127	1983	6,4	**HERVK**	191	841	22,7
**HERVL**	84	1082	7,7	**ERVL-B4**	142	1206	11,8
**MER4**	83	609	13,6	**HERVL**	132	1082	12,2
**ERVL-B4**	71	1206	5,9	**MER4**	126	609	20,7
**MER41**	56	442	12,6	**MER41**	77	442	17,4
**MER61**	40	355	11,2	**MER61**	76	355	21,4
**HARLEQUIN**	36	154	23,4	**HERV17**	53	227	23,3
