# Supplementary material for: Transcriptome Analysis of Human Endogenous Retroviruses at Locus-Specific Resolution in Non-Small Cell Lung Cancer
Source: Cancers (Basel). 2022 Sep 13;14(18):4433. doi: 10.3390/cancers14184433 (PMC9497127; doi:10.3390/cancers14184433)
Supplement: Supplementary file 1 [file cancers-14-04433-s001.zip › Table_S3.pdf]

LUAD					
1ST CLUSTER (148 HERV)			2ND CLUSTER (328 HERV)		
HERV SUB-FAMILY	N. DE HERVs	Perc. DE HERV family	HERV SUB-FAMILY	N. DE HERVs	Perc. DE HERV family
ERVL-E	17	11.5	HERVH	54	16.4
HERVH	14	9.4	ERVL-E	32	9.7
ERV3-16A3_I	13	8.8	HERVK	25	7.6
HERVL	11	7.4	MER4	23	7.0
HERVK	9	6.1	ERV3-16A3_I	21	6.4
HERVL18	8	5.4	ERVL-B4	20	6.1
ERVL-B4	7	4.7	MER41	13	3.9
MER4	6	4.1	HERVL	12	3.6
HARLEQUIN	5	3.4	MER61	10	3.0
HERV9	5	3.4	Harlequin	9	2.7

3RD CLUSTER (710 HERV)			4TH CLUSTER (211 HERV)		
HERV SUB-FAMILY	N. DE HERVs	Perc. DE HERV family	HERV SUB-FAMILY	N. DE HERVs	Perc. DE HERV family
HERVH	101	14.2	HERVH	44	20.8
ERVL-E	80	11.2	HERVK	23	10.9
HERVK	71	10.0	ERV3-16A3_I	15	7.1
ERV3-16A3_I	65	9.1	MER4	14	6.6
HERVL	36	5.1	HERVL	13	6.1
MER4	33	4.6	ERVL-E	10	4.7
ERVL-B4	32	4.5	ERVL-B4	8	3.8
MER41	25	3.5	MER41	8	3.8
HERVIP10F	16	2.2	HERVE	7	3.3
HERVL40	16	2.2	HERVL18	5	2.3
