# Supplementary material for: Transcriptome Analysis of Human Endogenous Retroviruses at Locus-Specific Resolution in Non-Small Cell Lung Cancer
Source: Cancers (Basel). 2022 Sep 13;14(18):4433. doi: 10.3390/cancers14184433 (PMC9497127; doi:10.3390/cancers14184433)
Supplement: Supplementary file 1 [file cancers-14-04433-s001.zip › Table_S4.pdf]

LUSC								
1ST CLUSTER (903 HERVS)			2ND CLUSTER (510 HERVs)			3rd CLUSTER (557 HERVs)		
HERV SUB-FAMILY	N. DE HERVs	Perc. DE HERV family	HERV SUB-FAMILY	N. DE HERVs	Perc. DE HERV family	HERV Sub-family	N. DE HERVs	Perc. DE HERV family
**HERVH**	113	12.5	**HERVH**	107	21.0	**HERVH**	78	14.0
**HERVK**	95	10.5	**ERVL-E**	46	9.0	**ERVL-E**	59	10.6
**ERVL-E**	79	8.7	**ERV3-16A3_I**	44	8.6	**ERV3-16A3_I**	58	10.4
**ERV3-16A3_I**	77	8.5	**MER4**	29	5.7	**HERVK**	49	8.8
**ERVL-B4**	59	6.5	**ERVL-B4**	28	5.5	**ERVL-B4**	33	5.9
**HERVL**	51	5.6	**HERVK**	28	5.5	**MER4**	29	5.2
**MER4**	41	4.5	**HERVL**	27	5.3	**HERVL**	27	4.8
**MER41**	33	3.6	**MER61**	18	3.5	**MER41**	19	3.4
**MER61**	28	3.1	**MER41**	15	2.9	**MER61**	19	3.4
**HERVL40**	23	2.5	**HERVE**	11	2.1	**HERV17**	15	2.7
